# The prevalence of complementary and alternative medicine use among Turkish patients with primary headache

**DOI:** 10.3906/sag-2102-234

**Published:** 2021-08-30

**Authors:** Büşra Sümeyye ARICA POLAT, Ayşe Çağlar SARILAR

**Affiliations:** 1 Department of Neurology, Gülhane Training and Research Hospital, Ankara Turkey; 2 Department of Neurology, School of Medicine, Erciyes University, Kayseri Turkey

**Keywords:** Complementary and alternative medicine, primary headache, prevalence

## Abstract

**Background/aim:**

The use of complementary and alternative medicine (CAM) is common in patients with primary headache. However, no study has been reported in which standardized modalities were questioned in a Turkish population. The aim of the present study was to investigate the frequency of CAM use and factors related to it in these patients.

**Materials and methods:**

Patients with a diagnosis of primary headache were included in this cross-sectional observational study. Demographic and disease specific characteristics were recorded. The use and effect of 15 CAM modalities were evaluated in accordance with the Traditional and Complementary Medicine Regulations. The patients were categorized into two groups according to their use of CAM procedures. Logistic regression analysis was further performed to assess the association between CAM use and related factors.

**Results:**

One hundred twenty patients [101 (84.2%) female, mean age 38.20 ± 12.24 years] were included. Use of CAM was reported in 33.3% of the patients. The most frequently used CAM modalities were phytotherapy (37.5%), cupping (27.5%), and chiropractic adjustment (17.5%). Compared with nonusers, CAM users showed a longer duration of disease (respectively mean 5.68 ± 4.96 years and 10.97 ± 8.57 years, p = 0.000). There were no differences with respect to age, sex, education, presence of systemic disease, headache subtypes, number of headache days in a month, or headache severity. Patients who underwent cupping reported that they benefited more than those who tried phytotherapy and multiple CAM methods (respectively 45.5%, 33.3%, 16.6%, p = 0.039). Subsequently, the logistic regression analysis showed a significant association between only disease duration and CAM use [respectively p value, OR (95% CI), and confidence intervals = 0.002 (1.143 (1.050–1.243)].

**Conclusion:**

Our results suggest that Turkish patients with primary headache, especially those with long disease duration, use CAM modalities. Larger population-based studies are required to clarify the safety and efficacy of these methods.

## 1. Introduction 

Primary headaches are headaches that are not associated with the central nervous system or other systemic diseases and they are classified under four main headings by the International Headache Society (IHA): migraine, tension-type headache (TTH), trigeminal autonomic cephalalgias, and other primary headache disorders [1]. 

While some patients with primary headache benefit from conventional medical treatments, many report that they did not obtain satisfactory relief or a lasting therapeutic effect, or that they had to discontinue the treatment due to the side effects of the recommended drugs [2]. For this reason, patients who have suffered from headaches for a long period and who have not benefited from the drugs they have taken are seeking complementary and/or alternative methods of treatment [2]. Complementary and alternative medicine (CAM) is defined as a diverse range of autonomous healthcare practices used for health maintenance, health promotion, disease prevention and for the treatment of ill-health. These practices can be used alone or in combination with conventional treatments [3]. In Turkey, practices are defined according to the government regulation concerning traditional medicine and CAM [4].

The prevalence of using CAM modalities for primary headaches is estimated to be 19%–82% worldwide [5]. This rate varies between cultures. CAM modalities are frequently used in combination with standard treatments for primary headaches in Turkey [6]. The prevalence of use of these modalities in migraine patients is reported to be 37% [7].

Very few studies have been performed on the prevalence of the use of CAM modalities in Turkish patients diagnosed with primary headache [6]. However, there is no study investigating this condition using standart CAM modalities defined by Turkish Ministry of Health. The first aim of the present study was to investigate the frequency of using CAM in patients diagnosed with primary headache. The second aim was to reveal the clinical characteristics of the patients with headache who were using these modalities and the factors associated with the application of CAM.

## 2. Materials and methods

### 2.1. Patient group

In this cross-sectional observational study, 120 patients (101 female, 19 male) who presented to the neurology outpatient clinic at a tertiary hospital and were diagnosed with primary headache according to the ICHD-III [1] classification were included. Primary headaches were classified as migraine, TTH, medication overuse headache (MOH) and mixed type headache which if the TTH and migraine diagnostic criteria were met. 

Migraine is a chronic headache that lasts 4–72 h; is usually unilateral, throbbing, and moderate or severe; and is characterized by recurrent attacks associated with nausea and/or vomiting or photophobia that increase with routine physical activities [1]. 

TTH is a common type of headache that is prominent in the bilateral occipital or frontal region, has a blunt and compressive character, is mild or moderate, does not interfere with routine daily activities, and does not have accompanying symptoms such as nausea or vomiting [8]. 

MOH, which is among the other primary headache disorders, is a chronic daily headache that occurs as a result of regular use of acute or symptomatic painkillers (simple analgesics, nonsteroidal antiinflammatory drugs, ergot preparations, and triptans) for 3 months or more due to primary headache [9]. 

The patients were evaluated by two neurologists and the primary headache classification was applied in accordance with the abovementioned criteria. Patients’ age, sex, educational status, presence of systemic disease, duration of illness, mean headache days in a month, and headache severity according to the visual analogue scale (VAS) were recorded. 

### 2.2. Clinical evaluation

The patients were evaluated in terms of the CAM modalities applied. In Turkey CAM modalities are dealt with in the Traditional and Complementary Medicine Regulation published in the Official Gazette No. 29158 dated 10.27.2014, which defines the following 15 modalities: 1. Acupuncture, 2. Apitherapy, 3. Phytotherapy, 4. Hypnosis, 5. Leech therapy (hirudotherapy), 6. Homeopathy, 7. Chiropractic adjustment, 8. Cupping, 9. Larval therapy, 10. Mesotherapy, 11. Prolotherapy, 12. Osteopathy, 13. Ozone therapy, 14. Reflexology, 15. Music therapy [4]. Soft tissue massages are evaluated under chiropractic adjustment. The patients were asked the following questions about these practices: (a) ‘Have you used one or more CAM modalities during any period of the disease after being diagnosed with primary headache? (b) If yes, which CAM modality did you use? (c) Did you benefit from the modality you used?. Patients who stated that they benefited from any modalities were asked to express this benefit as a percentage. The benefit was expressed as significant if the patient reported that he/she has benefited more than 50%, and as partial if it has been reported less. Patients who had tried at least one CAM modality were evaluated as (+) CAM application status.

The VAS was used to measure headache severity. The patients were asked to give a score according to the severity of pain, with 0 points if there was no pain and 10 points for the most severe pain.

Ethics committee approval was obtained for the study as well as approval from the patients concerning the use of their data for scientific purposes (Erciyes University Medical School Clinical Research Ethics Committee, 2020/613 approval code, 02.12.2020). The study was conducted in accordance with the principles of the Helsinki Declaration 2008. 

### 2.3. Statistical analysis

SPSS Statistics 21.0 sofware package (IBM Corporation, Armonk, NY, USA) was used for the analysis. Descriptive statistics were given as mean ± standard deviation (SD) for continuous data; and as count and proportion for categorical data. The distribution normality of the continuous variables was calculated with the Shapiro–Wilk test. We analysed the two groups with independent samples t test for the normally distributed variables and with the Mann–Whitney U Test for the nonnormally distributed variables to compare means. Categorical data were analysed with the chi-Square and Fisher’s exact tests. A logistic regression analysis was further performed to assess the effect of independent variables on CAM modality use. Data with p values greater 0.05 were considered not significant.

## 3. Results

One hundred twenty patients (101 female, mean age 38.2 ± 12.2 years; 19 males, mean age 31.8 ± 10.2 years) with a diagnosis of primary headache were included in the present study. Of these, 53.3% (n = 64) had migraine, 28.3% (n = 34) had TTH, 9.2% (n = 11) had MOH, and 9.2% (n = 11) had mixed type headache. Moreover, 33.3% (n = 40) of the patients stated that they used one or more CAM modality at least once due to their headaches.

The patients were divided into two groups: those using CAM (n = 40) and those not (n = 80). The two groups were similar in terms of age, sex, educational status, presence of systemic disease (diabetes mellitus, hypertension, hyperlipidemia, thyroid dysfunction, etc.), subtype of primary headache, mean number of headache days per month, and headache severity. The mean disease duration of the patients who tried CAM was longer than that of the patients that did not, and this difference was significant (mean 10.97 ± 8.57 years and 5.68 ± 4.96 years, respectively, p = 0.000) (Table 1).

**Table 1:** Demographic and clinical characteristics of headache patients using and not using CAM.

	CAM (+) (n = 40)	CAM (–)(n = 80)	p value
Age (years), mean (±SD)	39.5 (±11.8)	36.0 (±12.1)	0.132
Female sex n, (%)	33 (82.5)	68 (85)	0.724
Educational status Illiterate n, (%) Primary school n, (%) Middle school n, (%) High school n, (%) University and higher n, (%)	0 (0)12 (30) 6 (15) 18 (45) 4 (10)	4 (5)30 (37.5)11 (13.8)12 (28.8)12 (15)	0.285
Presence of systematic illness n, (%)	11 (27.5)	16(20)	0.354
Type of primary headache Migraine n, (%) TTH n, (%) Medication overuse headache n, (%) Mixed type headache n, (%)	23 (51.3)8 (20)4 (8.8)5 (7.5)	41 (57.5)26 (32.5)7 (10)6 (12.5)	0.486
Headache duration (years), mean (±SD)	10.97 ± 8.57	5.68 ± 4.96	0.000*
Headache frequency (days), mean (±SD)	9.75 ± 8.18	9.61 ± 7.97	0.930
VAS, mean (±SD)	7.62 ± 1.19	7.72 ± 1.86	0.785

SD, standard deviation; TTH, tension type headache; VAS, visual analogue scale; * p value < 0.05.

The most common CAM modalities were phytotherapy (drinking herbal infusions such as rosemary, bitter melon juice, lemon balm, centaury, and green tea and/or inhaling peppermint oil, lavender oil, and eucalyptus oil) and cupping (n = 15, 37.5%; n = 11, 27.5%, respectively). These were followed by chiropractic adjustment/massage, trying multiple modalities, and acupuncture (n = 7, 17.5%; n = 6, 15%; n = 1, 2.5%, respectively). The modalities that patients used more than once during their illness were acupuncture, cupping, hirudotherapy (leeches), and phytotherapy. Migraine patients most frequently used chiropractic adjustment/massage (n = 7, 10.9%). While TTH and mixed headache patients mostly used phytotherapy, MOH patients used cupping more frequently (n = 5, 14.7%; n = 32, 7.3%; n = 5, 14.7%, respectively) (Table 2). While 60% of the patients who used CAM stated that they did not benefit (n = 24), 30% reported significant benefits (n = 12) and 10% partial benefits (n = 4). Furthermore, 45.5% of the patients who underwent cupping, 33.3% of the patients who applied phytotherapy, and 16.6% of the patients who tried more than one CAM modality reported that they had benefited from these methods (p = 0.039). The percentage of total benefit seen for all methods averaged 51.25 % ± 19.27.

**Table 2:** CAM modality and frequency used for primary headaches.

	Migraine (N, %)	TTH (N, %)	MOH (N, %)	Mixed type headache (N, %)
Patients not using CAM	41 (64.1)	26 (76.5)	7 (63.6)	6 (54.5)
Phytotherapy	6 (9.4)	5 (14.7)	1 (9.1)	3 (27.3)
Cupping	4 (6.3)	3 (8.8)	3 (27.3)	1 (9.1)
Chiropractic adjustment/massage	7 (10.9)	0	0	0
Acupuncture	1 (1.6)	0	0	0
More than one modality (acupuncture, cupping, hirudotherapy, phytotherapy)	5 (7.8)	0	0	1 (9.1)

CAM, complementary and alternative medicine; TTH, tension type headache; MOH, medication overuse headache.

In addition, logistic regression was used to investigate the relationship between CAM modality use and age, sex, educational status, presence of systemic disease, and headache subtype, duration, frequency, and severity in patients diagnosed with primary headache. Age, sex, presence of systemic disease, and headache attack frequency and severity did not have a significant effect on CAM modality use. Similarly, educational status and subtype of headache were not related to the testing of these methods (p = 0.124 and p = 0.206, respectively). There was only an independent relationship between the duration of headache and the use of CAM [OR: 1.143 (95% CI: 1.050–1.243), p = 0.002]. The results of the regression analysis are given in detail in Table 3. 

**Table 3:** Results of logistic regression.

	OR	95% CI	p value
Age	1.033	0.975–1.096	0.269
Sex	0.661	0.180–2.419	0.531
Presence of systemic disease	0.671	0.196–2.296	0.525
Headache duration	1.143	1.050–1.243	0.002*
Headache frequency	1.014	0.946–1.086	0.703
VAS	0.836	0.641–1.091	0.188

OR, odds ratio; CI, confidence interval; VAS, visual analogue scale; *p value < 0.01.

## 4. Discussion 

The present study showed that one-third (33.3%) of patients with primary headache used at least one CAM modality. The ones most commonly used were phytotherapy and cupping. Moreover, no relationship was found between the use of CAM and the demographic characteristics of the patients or disease characteristics except headache duration. The duration of illness of the patients who tried CAM was longer than that in the patients who did not. While 60% of the patients stated that they had not benefited from these methods, the most useful method was cupping. 

Various studies have reported the frequency of using CAM for primary headaches at different rates in different populations. The frequency of CAM use was 32% in patients presenting to a headache clinic in the UK, whereas 82% of patients with headaches living in Austria and Germany used CAM [10,11]. In a broad-based national cross-sectional study conducted in the United States, 44.4% of patients with migraine and other chronic headaches used these methods [12]. In a Western society 62% of patients reported using CAM for primary chronic headaches [13]. In a Middle Eastern society, 69.5% of patients were found to have tried traditional methods for headaches [14]. The use of CAM in patients with headache in Turkey has been reported as 37% [7]. 

Differences in the prevalence of CAM use reported in the literature may arise for various reasons. In addition to cultural factors, the possibility of using these methods in patients presenting to a headache-specific neurology clinic may differ from that in the general population. In addition, the definitions of different CAM modalities adopted in studies may cause the results to vary [5]. It has been observed that most patients with chronic headaches prefer not to reveal their use of CAM modalities to their physicians due to fear of disapproval and not being taken seriously if they mentioned it [15]. This potential approach of the patients may cause differences in the prevalence of CAM use. In our study, 33.3% of patients with primary headache used CAM at least once during the course of the disease, similar to other study performed in Turkey. However, this percentage is lower than those reported in other studies. In our study, the patients who presented to a neurology clinic providing tertiary service were asked whether they used standard CAM modalities or not. Their preferred modalities (psychotherapy, vitamins, meditation and other relaxation techniques, etc.) other than CAM were excluded from the study. Moreover, patients may be reluctant to mention their use of these methods, and this might explain the difference in frequency of use.

In previous studies, acupuncture, massage, homeopathy, and chiropractic adjustment were reported as the modalities most commonly used for primary headaches [16,17]. Herbal treatments, chiropractic adjustment, massage, and body-mind therapies (such as meditation, biofeedback, and hypnosis) are frequently used for migraine and severe headaches [12,18]. Similarly, approximately one-fifth of TTH patients undergo chiropractic adjustment for their treatment [19]. Gaul et al. reported that 58.3% of patients used acupuncture, 46.1% massage, and 42.4% relaxation techniques for the prevention and treatment of headaches [11]. A study conducted in Kuwait showed that patients most frequently underwent cupping (65.6%) and massage (11.8%) for their headaches [14]. Among Turkish subjects, massage and phytotherapy are the most frequently used methods for primary headaches [6]. Another study found that Turkish migraine patients tried supplements and psychotherapy, but used standard CAM modalities less frequently, such as massage, phytotherapy, cupping, and acupuncture [7]. In our study, the most frequently used method among patients using CAM was phytotherapy. This was followed by cupping and chiropractic adjustment/soft tissue massage. These results differ from those of some previous studies, possibly due to the diversity in the definitions of CAM modalities. In addition, patients’ easier access to phytotherapy and massage may be why they prioritize these methods and use them more frequently. Again, cultural factors and religious beliefs can determine awareness of specific CAM practices such as cupping, the expected benefits, and the likelihood of being preferred. Similarly, although acupuncture is a method applied frequently in China and other Far Eastern countries, it is still not widely used in Turkey [6,20]. In our study, only three patients had tried acupuncture, which appears to be related to ease of accessibility as well as sociocultural factors.

It has been reported that 60%–73% of patients using CAM for primary headaches benefit from it [2,10]. In another study, the rate of satisfaction with these methods was 26.2% [14]. One-third of Turkish patients who used CAM stated that they only benefited from massage and that other methods did not work [6]. Similarly, 40% of our patients stated that they had benefited partially or significantly from the methods they tried. Unlike previously reported, it was observed that benefit from cupping therapy was the most common among all the methods in our study. The benefits of CAM modalities reported in studies are quite variable. This situation, which depends on the patients’ feedback, may be related to the personality patterns of the patients and their accompanying mood disorders, as well as the disease characteristics. In our study, these characteristics of the patients were not evaluated. It would be useful, however, to consider these factors when evaluating the perceived benefits from CAM. 

Application of CAM modalities to patients with primary headache is related to the characteristics of the patient and the disease. Advanced age, long duration of illness, presence of other illnesses other than headache such as anxiety and joint and back pain, and lifestyle characteristics predict CAM use [7,11,14,15]. Apart from these, it is known that women, married people, those with higher education and income levels, and those with more monthly headache days and hospital admissions use CAM modalities more frequently [5,11, 19, 21–23]. No relationship was found with the type or severity of headaches [6,7,11]. There are also studies showing that the use of CAM is not related to sex, age, education level, income level, or marital status [7,14]. Educational background results in some CAM modalities being known about, but it does not affect their use [6]. In our study, the duration of the disease affected the use of CAM, in line with the literature. However, no other patient or disease characteristics were shown to predict the use of CAM. 

To the best of our knowledge, the present study is the first to investigate the frequency of use of standard CAM practices for primary headaches in Turkish subjects. However, the study has some limitations. The patients were selected from among those who presented to a tertiary neurology outpatient clinic and there was a higher rate of patients with a diagnosis of migraine or TTH than the rate reported in the community. In addition, mood disorders that may affect the benefits patients received from CAM were not evaluated using objective measurements. The relatively small number of patients included is another limitation. 

In conclusion, use of CAM is frequent in Turkish patients with primary headache and is associated with long disease duration. The most frequently used CAM modalities are phytotherapy, cupping, and chiropractic adjustment/massage. CAM therapies are perceived as partially or significantly beneficial by 40% of users. The prevalence, type of CAM modalities, and benefits of these procedures may vary between different cultures. There is a need for randomized, controlled, large-scale studies investigating the use of CAM and related factors in the treatment and prevention of primary headaches in the general population. 
